# Impact of mechanical ventilation on the daily costs of ICU care: a systematic review and meta regression

**DOI:** 10.1017/S0950268819001900

**Published:** 2019-12-05

**Authors:** Klaus Kaier, Thomas Heister, Edith Motschall, Philip Hehn, Tobias Bluhmki, Martin Wolkewitz

**Affiliations:** 1 Institute of Medical Biometry and Statistics, Faculty of Medicine and Medical Center – University of Freiburg, Freiburg, Germany; 2 Institute of Statistics, Ulm University, Ulm, Germany

**Keywords:** Added costs, ICU costs, mechanical ventilation, review, ventilator-associated pneumonia

## Abstract

The impact of mechanical ventilation on the daily costs of intensive care unit (ICU) care is largely unknown. We thus conducted a systematic search for studies measuring the daily costs of ICU stays for general populations of adults (age ≥18 years) and the added costs of mechanical ventilation. The relative increase in the daily costs was estimated using random effects meta regression. The results of the analyses were applied to a recent study calculating the excess length-of-stay associated with ICU-acquired (ventilator-associated) pneumonia, a major complication of mechanical ventilation. The search identified five eligible studies including a total of 54 766 patients and ~238 037 patient days in the ICU. Overall, mechanical ventilation was associated with a 25.8% (95% CI 4.7%–51.2%) increase in the daily costs of ICU care. A combination of these estimates with standardised unit costs results in approximate daily costs of a single ventilated ICU day of €1654 and €1580 in France and Germany, respectively. Mechanical ventilation is a major driver of ICU costs and should be taken into account when measuring the financial burden of adverse events in ICU settings.

## Background

Intensive care consumes a large proportion of healthcare resources. Days in intensive care are substantially more costly than general ward days in hospitals due to increased resource utilization and labour intensity. One major driver of these costs is mechanical ventilation.

Adverse events occurring during hospitalization add a substantial further burden to the healthcare system. The most prevalent hospital-onset conditions are hospital-acquired infections, which absorb substantial resources in hospitals and often require costly treatment [1]. For patients under mechanical ventilation, one of the infections commonly acquired in the hospital is ventilator-associated pneumonia (VAP).

The major aim of this review is to determine the relative impact of mechanical ventilation on the daily costs of intensive care unit (ICU) care. We believe that this information is crucial in a number of contexts but has been neglected given the unavailability of reliable estimates.

We therefore conducted a systematic review regarding the costs of an ICU day and the proportion of these costs attributable to mechanical ventilation and quantified the relative increase in the daily costs using meta regression. In order to illustrate the practical relevance of these findings, we then determine the economic burden of VAP using recent cost information from France and Germany.

## Methods

### Literature search

On 24 March 2017 we searched MEDLINE via Wolters Kluwer's search interface Ovid (indexed and non-indexed databases), Web of Science via Thomson Reuters, (now Clarivate Analytics), CINAHL via EBSCOhost and the NHS Economic Evaluation Database (NHS EED) via Centre for Reviews and Dissemination, University of York. We performed the searches restricted to the English language and publication year 2000 to the update status at date of search (2017).

The search strategies varied by the database used. For details of the search strategies in all databases, see Supplementary material (Appendix). The searches returned 2839 studies of potential interest. After elimination of duplicates, 2072 studies remained.

### Eligibility criteria

**Fig. 1.**
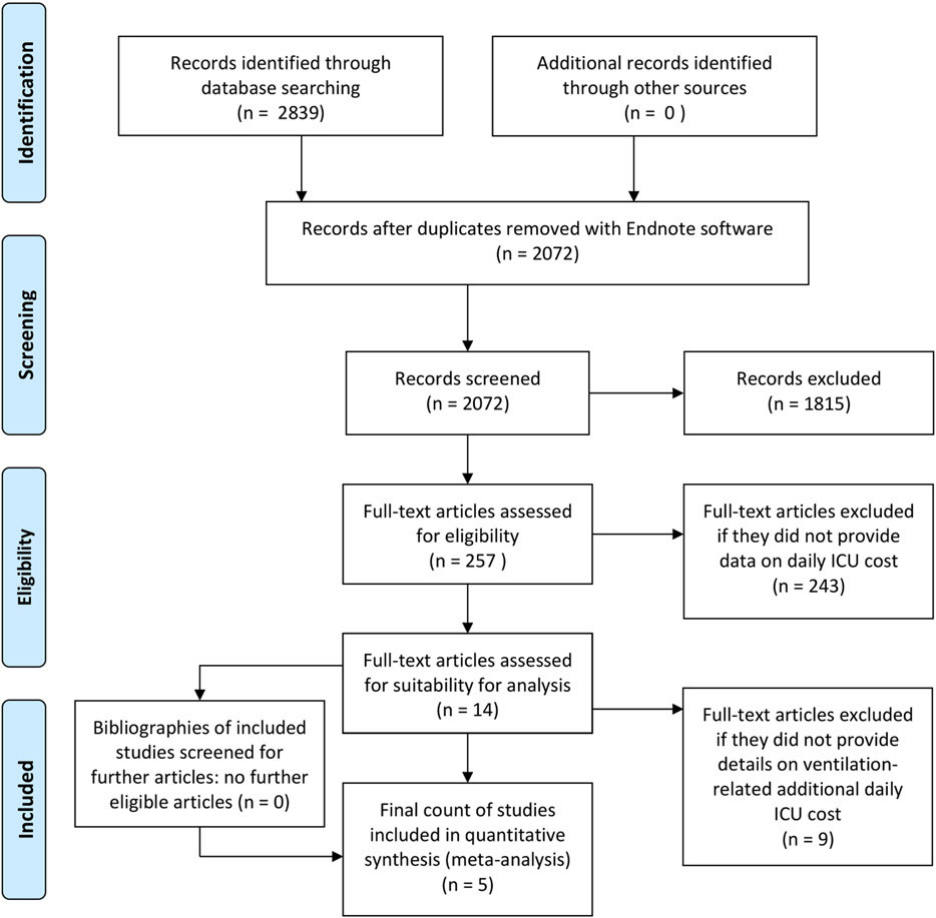
PRISMA flow diagram.

Studies were eligible if they gave data for the daily costs of ICU stays for general populations of adults (age ⩾ 18 years) and the added costs of mechanical ventilation. Screening of titles and abstracts found 257 potentially relevant papers. We were able to access full-texts of all articles of interest. Of these, 14 articles provided data on the daily costs of ICU stays, but only five articles [2–6] fulfilled our inclusion criteria of also providing information regarding the added costs of mechanical ventilation (Fig. 1: PRISMA-chart). No additional relevant articles were found in the reference lists of the 14 articles providing data on the daily costs of ICU stay.

### Data extraction

Two authors (KK and PH) checked the titles and abstracts of all potentially relevant English articles, and the full text of these was obtained. The same authors independently checked all the full-text articles for eligibility, and author KK extracted clinical data from the full texts. Details on the number of patients, ICU days, daily ICU costs and corresponding measures of uncertainty (standard deviations or 95% confidence intervals) were derived from the studies for ICU patients currently under mechanical ventilation or not. If necessary, patient days were derived from the proportion of patients ventilated and mean/median length of ICU stay (LoS). In the case of no reported variances, a standard deviation of 20% was imputed. In two cases (Fig. 1) it was assumed that ventilation takes place during 50% of total ICU stay.

### Data analysis

Daily ICU costs were log transformed along with their standard errors and analysed using random effects meta regression (command metareg [7–9] in Stata). As the studies' base years and currencies vary, an additional fixed effect on the study level was added to the meta regression. For the individual studies, *t*-tests based on summary data (command ttesti in Stata) were applied on log transformed daily costs. As the latter costs were used in all analyses, resulting coefficients were exponentiated in order to show the relative effect of ventilation on the daily ICU costs. Please note that the relative increase is always taking place within a country and year, therefore making adjustment for inflation and/or currency exchange rates unnecessary.

### Application

The results of the different analyses were applied to recent results by Bluhmki *et al*. who calculated the excess LoS associated with ICU-acquired pneumonia [10]. Due to methodological issues and/or lack of cost data, many studies analyse the burden of ICU-acquired pneumonia regarding the endpoint LoS but aim to express the results on a pecuniary scale as well. In comparison with the endpoint costs, analysing LoS is advantageous as it enables accounting for the time-dynamic pattern of the exposure. In line with Bluhmki *et al*. [10] excess LoS was derived using a multistate methodology, which accounted for the time-dynamic pattern of both VAP as well as ventilation status. Their precise formulation further allows decomposing the excess LoS into extra days spent under, and not under ventilation. We emphasise that ignoring the time-dependency of VAP would lead to a substantial overestimation of extra days spent in the ICU [11 12]. On the one hand, this decomposition expresses the disease burden and a patient's quality status in more detail but it is also highly relevant from an economic point of view, because ventilation is known to be a major cost driver in the ICU. Thus, we combine our results with summary measures provided by Bluhmki *et al*. [10] in order to obtain a more precise estimate of the additional costs associated with VAP.

For all statistical analyses Stata Version 14.0 (Stata Corp, College Station, Texas, USA) was used.

## Results

**Fig. 2.**
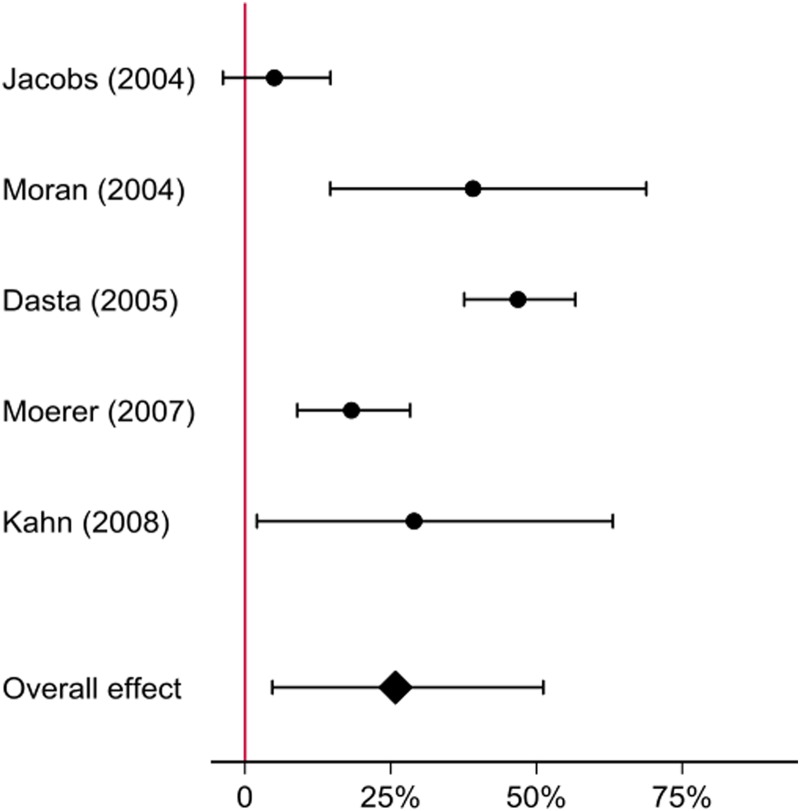
Relative increase in daily ICU costs due to ventilation. For the individual studies, *t*-tests are applied on log transformed daily costs while the overall effect is calculated using random effects meta regression [8]. All resulting coefficients are exponentiated in order to show the relative effect of ventilation on the daily ICU costs.

The selection of studies is summarised in Figure 1. Ultimately, five studies were included in the analysis representing a total of 54 766 patients and ~238 037 patient days (Table 1). Four studies included detailed cost figures calculated from the hospital perspective [3–6], while the other derived daily charges and multiplied these by hospital specific cost-to-charge ratios in order to give the hospital perspective [2]. As shown in Figure 2, there was strong variability in the relative effect of mechanical ventilation across studies but overall, mechanical ventilation was associated with a 25.8% (95% CI 4.7%–51.2%) increase in the daily costs of ICU care.

**Table 1.** Overview of studies

Author	Year	Country	Number of patients	Patient days non-ventilated	Patient days ventilated	Daily costs, non-ventilated	Daily costs, ventilated	Base year	Detailed cost figures from the hospital perspective available?
Jacobs	2004	UK	193	910	480^a^	GBP 455	GBP 587	2000–1	Yes
Moran	2004	Australia	1333	2933	2266	AU$ 1616	AU$ 1911	1991	Yes
Dasta	2005	USA	51 009	117 275	104 104	USD 3250	USD 4772	2002	No^b^
Moerer	2007	Germany	453	2154	567^a^	EUR 680	EUR 946	2003	Yes
Kahn	2008	USA	1778	5879	1469^a^	USD 2104	USD 2210	2005–6	Yes

PD = Patient days.

a Patient days derived from the proportion of patients ventilated and mean/median LoS. In two studies (Kahn and Jacobs) it was also assumed that ventilation takes place during 50% of total ICU stay.

b Daily costs were estimated by multiplying daily hospital charges by hospital specific cost-to-charge ratios.

### Application

Bluhmki *et al*. [10] reported a total extra LoS due to VAP of 3.52 days (95% CI 2.46%–4.59%) and found that the ‘excess LoS associated VAP is mainly triggered by excess LoS under ventilation, whereas the excess LoS under non-ventilation is almost negligible’. This group also conducted a more complex analysis allowing for a decomposition of excess LoS under ventilation and non-ventilation. For illustrative purposes and simplicity, we have used the summary measure based on the multistate model not distinguishing between ventilation and non-ventilation.

The study of Lefrant *et al*. [13] provided standardised unit costs (€1425 per ICU day, in 2008 Euros) for patients hospitalised in French ICUs and 65% of their patients received mechanical ventilation. If we further assume that, among these patients, ventilation took place during 50% of ICU stay, 32.5% of all patients days were ventilated ICU days. Combining these results (€1425) with the ventilation-related cost increase of 25.8%, results in daily costs of a single ventilated ICU day of €1654. Unfortunately, the duration of ventilation is not reported in the study making an assumption regarding the mean duration of ventilation necessary. For simplicity, we have assumed that ventilation takes place during 50% of ICU stay. The resulting 32.5% (0.5 × 0.65) share of ventilated ICU days may then be used to combine overall daily ICU costs (€ 1425, including both ventilated and non-ventilated ICU days) with our estimate regarding the ventilation-related cost increase (we have used the formulas €1425 = 0.325 × Cost_ventilated + (1−0.325) × Cost_(non-ventilated) and Cost_ventilated = 1.258 × Cost_(non-ventilated)). However, if we instead assume that ventilation takes place during 75% of ICU stay, the cost of a single ventilated ICU day changes slightly to €1592. Further combination of this cost figure with Bluhmki's data [10] (3.52 days of excess LoS) then results in the financial burden of a single VAP of €5822 (95% CI €4012–€ 7632).

Bock *et al*. provide standardised unit costs (€1338 per ICU day, in 2011 Euros) for patients hospitalised in German ICUs [14]. Assuming that 50% of patients are ventilated and ventilation takes place during 50% of their respective ICU stay, we may combine these estimates (€1338) with the ventilation-related cost increase (25.8%) to obtain daily costs of a single ventilated ICU day of €1581. Further combination of this cost figure with Bluhmki's results [7] (3.52 days of excess LoS) then results in the financial burden of a single VAP of €5565 (95% CI €3881–€7249).

## Discussion

Intensive care absorbs a large proportion of health care spending in industrialised countries [15]. Reliable information on the magnitude and variability of these costs is essential in order to guide spending decisions and as a starting point in identifying possible saving potentials. This is especially topical in light of variability between countries in resource use and concerns about demand elasticity [16–19].

The results of the systematic review and meta regression analysis showed that mechanical ventilation is associated with a substantial increase in the daily costs of ICU care. Moreover, we observed a strong variability in the relative effect of mechanical ventilation across studies. However, the number of appropriate studies was rather limited and we were unable to find relevant more recent studies. As technology, procedures and indications for ventilation have changed in the meantime, our findings may be limited in scope and further studies on this topic are needed.

In the estimation of the additional LoS of adverse events during hospitalization, it is crucial to treat the exposure as time-varying to avoid a time-dependent bias [11 12]. This bias occurs when it is implicitly assumed that the conditions are already present on admission and leads to an overestimation of the incremental effect [20]. Bluhmki *et al*. were aware of this fact and correctly estimated the excess LoS associated with VAP using multistate models accounting for the time-dynamics of ventilation status and VAP. At the same time, Bluhmki *et al*. [10] considered that ignoring the time-dependency of VAP would lead to an excess LoS of 15 days, which represents a substantial overestimation in comparison with the more precise effect estimate of 3.52 days. This might also be the case in other studies, when cost figures are available, but ignoring the time-dependency of the exposure might have led to a substantial overestimation of the true effect [12 20–22].

Our cost calculations were based on the simplifying assumption that the entire excess LoS can be attributed to ventilation; thus, we may have slightly overestimated the costs. However, we refer to the result that this excess seems to be mainly triggered by ventilation [10]. While LoS may be of intrinsic interest as an indicator for the burden on the patient level, it is a rather incomplete proxy for costs as the care intensity of the respective patients is not taken into account. Nonetheless, many studies multiply the extra LoS derived from multistate models with time fixed average daily costs, or standardised unit costs [23]. Not taking care intensity into account, however, leads to an underestimation of the costs, as post-infection daily costs are higher than average daily costs. Only a few studies use more accurate average cost per intensive care day, however only to estimate the financial burden of hospital-acquired infections, so they could not be used in our analysis [24–26]. For severe infections such as pneumonia, however, ventilation is a major driver of ICU cost and should be taken into account when measuring the financial burden of adverse events in ICU settings.

## Conclusions

Our study contributes to a more detailed understanding and measuring of the costs of intensive care and mechanical ventilation by providing first estimates and discussing methodological particularities. Being an area with relatively little empirical evidence to date, more studies on the daily costs of mechanical ventilation and intensive care are duly needed.

## Supplementary material

For supplementary material accompanying this paper visit https://doi.org/10.1017S0950268819001900. click here to view supplementary material

## Data

The study is based on publicly available data.
