# Supplementary material for: Impact of mechanical ventilation on the daily costs of ICU care: a systematic review and meta regression
Source: Epidemiol Infect. 2019 Dec 5;147:e314. doi: 10.1017/S0950268819001900 (PMC7003623; doi:10.1017/S0950268819001900)
Supplement: Supplementary file 1 [file S0950268819001900sup001.zip › S0950268819001900sup001/S1_Appendix_databases.docx]

# *Epidemiology and Infection*

# **Impact of mechanical ventilation on the daily costs of ICU care: a systematic review and meta regression**

Klaus Kaier; Thomas Heister; Edith Motschall; Philip Hehn; Tobias Bluhmki; Martin Wolkewitz, on behalf of COMBACTE-MAGNET Consortium (<www.combacte.com>)

**Supplementary Material**

S1: Databases searched

ICU – daily costs / Searcher: Edith Motschall

| Database | Host/Interface | Search date | Update status | Results |
| --- | --- | --- | --- | --- |
| Medline | Wolters Kluwer/Ovid | 2017-03-24 | 1946 to March Week 3 2017 | 1397 |
| MEDLINE Daily Update |  |  | March 23, 2017 |  |
| MEDLINE In-Process & Other Non-Indexed Citations |  |  | March 23, 2017 | 194 |
| MEDLINE Epub Ahead of Print |  |  | March 23, 2017 |  |
| Web of Science:  Science Citation Index,  Social Science Citation Index | Thomson Reuters (now Clarivate Analytics)/Web of Science |  | 2017-03-23 | 347 |
| CINAHL | EBSCO |  | Not given | 790 |
| NHS Economic Evaluation Database (NHS EED) | Centre for Reviews and Dissemination, University of York | 24.3.2017 | 2010- 31st March 2015 (updates ceased) | 111 |
|  |  |  | Total with dupl | 2839 |
|  |  |  | **Total without duplicates** (checked with Endnote software and manually) | **2072** |
