# Supplementary material for: Impact of mechanical ventilation on the daily costs of ICU care: a systematic review and meta regression
Source: Epidemiol Infect. 2019 Dec 5;147:e314. doi: 10.1017/S0950268819001900 (PMC7003623; doi:10.1017/S0950268819001900)
Supplement: Supplementary file 1 [file S0950268819001900sup001.zip › S0950268819001900sup001/S2_Appendix_strategies.docx]

# *Epidemiology and Infection*

# Impact of mechanical ventilation on the daily costs of ICU care: a systematic review and meta regression

Klaus Kaier; Thomas Heister; Edith Motschall; Philip Hehn; Tobias Bluhmki; Martin Wolkewitz, on behalf of COMBACTE-MAGNET Consortium (<www.combacte.com>)

**Supplementary Material**

S2: Search strategies

Topic: ICU – daily costs (searcher: Edith Motschall)

Database(s):

Via Wolters Kluwer, search interface Ovid:

Indexed part of Medline:

**MEDLINE** 1946 to March Week 3 2017,

**MEDLINE Daily Update** March 23, 2017

Search date (yyyy-mm-dd): 2017-03-24

| **#** | **Searches** | **Comments** | **Results** |
| --- | --- | --- | --- |
| 1 | exp Intensive Care Units/ | Facet Crit Care or ICU: MeSH with any subheading | 67945 |
| 2 | Critical Care/ |  | 45313 |
| 3 | 1 or 2 |  | 104669 |
| 4 | Hospital Costs/ | Facet cost: MeSH | 9432 |
| 5 | hospital charges/ |  | 2712 |
| 6 | exp "Costs and Cost Analysis"/ |  | 209044 |
| 7 | economics.fs. | Economics as floating subheading (for any MeSH) | 382634 |
| 8 | or/4-7 | Facet costs | 438923 |
| 9 | 3 and 8 | ICU and cost (MeSH) 🡪too many hits 🡪 restriction with #10 | 5160 |
| 10 | (day or days or daily).ti,ab,kf. | Facet daily as textwords | 1749282 |
| 11 | 9 and 10 | ICU and cost (MeSH) and daily (Textword):  **1. partial result** | 1469 |
| 12 | exp Intensive Care Units/ec | Facet Crit Care or ICU: MeSH with subheading economics | 1999 |
| 13 | Critical Care/ec |  | 1261 |
| 14 | 12 or 13 |  | 2970 |
| 15 | or/4-6 | Facet cost: MeSH | 210640 |
| 16 | 14 and 15 | Facet ICU with subheading econ and facet cost (MesH):  **2. partial result** | 2094 |
| 17 | exp *Intensive Care Units/ec | Facet Crit Care or ICU: MeSH with subheading economics and focus (main aspect) 🡪 specific:  **3. partial result** | 883 |
| 18 | *Critical Care/ec |  | 509 |
| 19 | 17 or 18 |  | 1287 |
| 20 | (intensive care or critical care or intensive treatment* or intensive therap* or ICU or ICUs).ti. | Facet ICU: textwords in title (specific) | 45805 |
| 21 | (cost or costs or reimburs* or re-imburs* or charge* or expenditure*).ti. | Facet costs: textwords in title (specific) | 110203 |
| 22 | 20 and 21 | Facet ICU and costs: textwords in title (specific):  **4. partial result** | 701 |
| 23 | ((cost or costs or reimburs* or re-imburs* or charge* or expenditure*) adj6 (day or days or daily)).ti,ab,kf. | Facet cost and daily within 6 words (textwords in title, abstract, keywords) | 10509 |
| 24 | 20 and 23 | Facet ICU (textwords in title) and costs and daily within 6 words in ti,ab,keyword  **5. partial result** | 277 |
| 25 | 11 or 16 or 19 or 22 or 24 | OR-combination if partial results | 3281 |
| 26 | (review or comment or editorial or letter).pt. | Excluding publication types of #26 | 3599794 |
| 27 | 25 not 26 |  | 2635 |
| 28 | 27 and eng.la. and 20*.yr. | Limit to english and year >=2000 | 1443 |
| 29 | remove duplicates from 28 | Elimination of duplicates.  **Final result of indexed part of Medline** | 1397 |

Legend:

/ = Medical Subject Heading (MeSH)

/ec = MeSH with subheading economics

economics.fs. = economics as floating subheading (for any MeSH)

ti,ab,kf = title, abstract, keyword

adj6 = within 6 words in any order

exp = explode MeSH

exp * = explode MeSH as focus (main topic)

.fs. = floating subheading

.la. = language

.pt. = publication type

.ti. = title

.yr. = publication year

* = truncation (variable ending of textword)

Non-indexed part:

**MEDLINE In-Process & Other Non-Indexed Citations** March 23, 2017,

**MEDLINE Epub Ahead of Print** March 23, 2017
Search date 2017-03-24

| **#** | **Searches** | **Comments** | **Results** |
| --- | --- | --- | --- |
| 1 | (intensive care or critical care or intensive treatment* or intensive therap* or ICU or ICUs).ti. | Facet ICU: textwords in title (specific) | 5786 |
| 2 | (cost or costs or reimburs* or re-imburs* or charge* or expenditure*).ti,ab,kf. | Facet costs: textwords in title, abstract, keywords | 134092 |
| 3 | 1 and 2 | ICU in title and costs in title, abstracts, keyword | 404 |
| 4 | (day or days or daily).ti,ab,kf. | Facet daily in title, abstract, keywords | 189281 |
| 5 | 3 and 4 | ICU in title and costs in title, abstracts, keyword and daily in title, abstracts, keyword:  **1. partial result** | 162 |
| 6 | (cost or costs or reimburs* or re-imburs* or charge* or expenditure*).ti. | Facet costs: textwords in title | 27580 |
| 7 | 1 and 6 | Facets ICU and costs in title (specific):  **2. partial result** | 90 |
| 8 | 5 or 7 | OR-combination of partial results | 213 |
| 9 | (review or comment or editorial or letter).pt. | Exclusion of publication types | 200260 |
| 10 | 8 not 9 |  | 200 |
| 11 | 10 and eng.la. and 20*.yr. | Restriction to English and year >=2000:  **Final result of non-indexed part** | 194 |

Legend:

ti,ab,kf = title, abstract, keyword

.la. = language

.pt. = publication type

.ti. = title

.yr. = publication year

* = truncation (variable ending of textword)

Via Thomson Reuters (now Clarivate Analytics): Web of Science databases:

**Science Citation Index Expanded** (SCI-EXPANDED): 1945-present

**Social Sciences Citation Index** (SSCI): 1956-present

Data last updated: 2017-03-23

Search date: 2017-03-24

| **Set** | **Results** |  | **Comments (read from bottom to top)** |
| --- | --- | --- | --- |
| # 7 | [347](http://apps.webofknowledge.com/summary.do?product=WOS&doc=1&qid=16&SID=R2TniiqU7SDgxj1XT2N&search_mode=AdvancedSearch&update_back2search_link_param=yes) | (#6) *AND* **LANGUAGE:** (English) *AND* **DOCUMENT TYPES:** (Article)  Indexes=SCI-EXPANDED, SSCI Timespan=2000-2017 | Restriction to Englisch, Articles and year >=2000:  **Final result** |
| # 6 | [1,068](http://apps.webofknowledge.com/summary.do?product=WOS&doc=1&qid=14&SID=R2TniiqU7SDgxj1XT2N&search_mode=CombineSearches&update_back2search_link_param=yes) | #5 OR #3  Indexes=SCI-EXPANDED, SSCI Timespan=All years | OR-combination of partial results |
| # 5 | [272](http://apps.webofknowledge.com/summary.do?product=WOS&doc=1&qid=13&SID=R2TniiqU7SDgxj1XT2N&search_mode=CombineSearches&update_back2search_link_param=yes) | #4 AND #1  Indexes=SCI-EXPANDED, SSCI Timespan=All years | Facet ICU in title and daily and (within 6 words) costs in title, abstract, keywords .  **2. partial result** |
| # 4 | [12,299](http://apps.webofknowledge.com/summary.do?product=WOS&doc=1&qid=12&SID=R2TniiqU7SDgxj1XT2N&search_mode=AdvancedSearch&update_back2search_link_param=yes) | TS=((day or days or daily) near/6 (cost or costs or reimburs* or "re-imburs*" or charge* or expenditure*))  Indexes=SCI-EXPANDED, SSCI Timespan=All years | Facet daily and costs within 6 words in title, abstract, keywords |
| # 3 | [957](http://apps.webofknowledge.com/summary.do?product=WOS&doc=1&qid=3&SID=R2TniiqU7SDgxj1XT2N&search_mode=CombineSearches&update_back2search_link_param=yes) | #2 AND #1  Indexes=SCI-EXPANDED, SSCI Timespan=All years | Facet ICU and costs: words in title.  **1. partial result** |
| # 2 | [349,765](http://apps.webofknowledge.com/summary.do?product=WOS&doc=1&qid=2&SID=R2TniiqU7SDgxj1XT2N&search_mode=AdvancedSearch&update_back2search_link_param=yes) | TI= (cost or costs or reimburs* or "re-imburs*" or charge* or expenditure*)  Indexes=SCI-EXPANDED, SSCI Timespan=All years | Facet costs: words in title |
| # 1 | [51,603](http://apps.webofknowledge.com/summary.do?product=WOS&doc=1&qid=1&SID=R2TniiqU7SDgxj1XT2N&search_mode=AdvancedSearch&update_back2search_link_param=yes) | TI=("intensive care" or "critical care" or "intensive treatment*" or "intensive therap*" or ICU or ICUs)  Indexes=SCI-EXPANDED, SSCI Timespan=All years | Facet ICU: words in title |

Legend:

TI = title

TS=Topic: Searches in the following fields: Title, Abstract, Author Keywords, Keywords Plus®

near/6 = within 6 words in any order

* = truncation

**CINAHL (via EBSCOhost)**

Search date 2017-03-24

Update Status: not given

Advanced Search screen

Limiters - Published Date: 20000101-20171231
Search modes - Boolean/Phrase

| \| **#** \| **Query** \| **Comment**  Read from bottom to top \| **Results** \| \| --- \| --- \| --- \| --- \| \| S20 \| s16 not s17  Limiters - Published Date: 20000101-20171231,  English \| Limit to year 2000-2017, English.  **Final result** \| 790 \| \| S19 \| s16 not s17  Limiters - Published Date: 20000101-20171231 \| Limit to year 2000-2017 \| 803 \| \| S18 \| s16 not s17 \| Exclusion of publication types \| 1,076 \| \| S17 \| PT (editorial OR review OR comments OR letters) \| 282,798 \| \| S16 \| S7 OR S10 OR S13 OR S15 \| OR-combination of partial results \| 1,172 \| \| S15 \| S11 AND S14 \| Facet ICU in title and daily and (within 6 words) costs in all text.  **4. partial result** \| 92 \| \| S14 \| TX (day or days or daily) N6 (cost or costs or reimburs* or "re-imburs*" or charge* or expenditure*) \| Facet daily and costs within 6 words in all text \| 3,118 \| \| S13 \| S11 AND S12 \| ICU and costs: Textwords in title  **3. partial result** \| 327 \| \| S12 \| TI (cost or costs or reimburs* or re-imburs* or charge* or expenditure*) \| Facet costs: Textwords in title \| 33,865 \| \| S11 \| TI (intensive care or critical care or intensive treatment* or intensive therap* or ICU or ICUs) \| Facet ICU: Textwords in title \| 25,402 \| \| S10 \| S8 OR S9 \| Facet ICU: MeSH with subheading economics  **2. partial result** (S10) \| 715 \| \| S9 \| (MH "Critical Care+/EC") \| 405 \| \| S8 \| (MH "Intensive Care Units+/EC") \| 380 \| \| S7 \| S3 AND S6 \| ICU and costs (MeSH)  **1.partial result** \| 481 \| \| S6 \| S4 OR S5 \| Facet costs: MeSH \| 12,928 \| \| S5 \| (MH "Costs and Cost Analysis") \| 9,922 \| \| S4 \| (MH "Health Facility Costs") OR (MH "Nursing Costs") \| 3,396 \| \| S3 \| S1 OR S2 \| Facet ICU: exploded MeSH \| 41,572 \| \| S2 \| (MH "Critical Care+") \| 15,497 \| \| S1 \| (MH "Intensive Care Units+") \| 30,881 \| |
| --- | --- | --- | --- | --- | --- | --- | --- | --- | --- | --- | --- | --- | --- | --- | --- | --- | --- | --- | --- | --- | --- | --- | --- | --- | --- | --- | --- | --- | --- | --- | --- | --- | --- | --- | --- | --- | --- | --- | --- | --- | --- | --- | --- | --- | --- | --- | --- | --- | --- | --- | --- | --- | --- | --- | --- | --- | --- | --- | --- | --- | --- | --- | --- | --- | --- | --- | --- | --- | --- | --- | --- | --- | --- | --- | --- | --- | --- |
|  |

Legend:

MH = MeSH

MH *term*+ = exploded MeSH

/EC = MeSH with subheading economics

TI = title

TX = all text

PT = publication type

N6 = within 6 words in any order

* = truncation

Database: **NHS Economic Evaluation Database (NHS EED)**

<https://www.crd.york.ac.uk/CRDWeb/HomePage.asp>

From: Centre for Reviews and Dissemination, University of York

Search date: 2017-03-24

Time span: 2000 - March 2015 (update ceased)

| Search  No | Query | Comments | Hits |
| --- | --- | --- | --- |
| 1 | (cost or costs or reimburs* or re-imburs* or charge* or expenditure*):TI IN NHSEED FROM 2000 TO 2015 | Facet cost in title | 9395 |
| 2 | (intensive care or critical care or intensive treatment* or intensive therap* or ICU or ICUs):TI IN NHSEED FROM 2000 TO 2015 | Facet ICU in title | 131 |
| 3 | #1 AND #2 | Facet ICU and cost: Textwords in title. | 63 |
| 4 | ((cost or costs or reimburs* or re-imburs* or charge* or expenditure*) near (day or days or daily)) IN NHSEED FROM 2000 TO 2015 | Facet daily and costs within 6 words in all fields | 650 |
| 5 | #2 AND #4 | Facet ICU in title and daily and (within 6 words) costs in all text. | 17 |
| 6 | #3 OR #5 | OR-combination of partial results from #3, #5:  **1. partial result** | 71 |
| 7 | MeSH DESCRIPTOR intensive care units EXPLODE ALL TREES WITH QUALIFIER EC IN NHSEED | Facet ICU: MeSH with subheading economics. | 148 |
| 8 | MeSH DESCRIPTOR critical care EXPLODE ALL TREES WITH QUALIFIER EC IN NHSEED |  | 102 |
| 9 | #7 OR #8 |  | 233 |
| 10 | MeSH DESCRIPTOR Hospital Costs IN NHSEED | Facet costs: MeSH | 1020 |
| 11 | MeSH DESCRIPTOR Hospital Charges IN NHSEED |  | 205 |
| 12 | MeSH DESCRIPTOR Costs and Cost Analysis EXPLODE ALL TREES IN NHSEED |  | 15030 |
| 13 | #10 OR #11 OR #12 |  | 15119 |
| 14 | #9 AND #13 | Facet ICU MeSH with subheading economics and Costs (MeSH) | 206 |
| 15 | (day or days or daily) IN NHSEED FROM 2000 TO 2015 | Facet daily: Textwords in all fields | 3413 |
| 16 | #14 AND #15 | Facet ICU MeSH with subheading economics and Costs (MeSH) and textwords daily in all fields.  **2. partial result** | 62 |
| 17 | #6 OR #16 | OR-combination of partial results. **Final result** | 111 |

Legend:

Near = proximity operator: within 6 words in any order

* = truncation

TI = title
